# Infección por *Trypanosoma cruzi* en mujeres puérperas y sus neonatos en Barcelona, estado Anzoátegui, Venezuela

**DOI:** 10.7705/biomedica.4606

**Published:** 2019-12-30

**Authors:** Norielis del Carmen Zabala, Mariolga Berrizbeitia, Alicia Jorquera, Jéssicca Rodríguez, Leomery Romero

**Affiliations:** 1 Posgrado en Biología Aplicada, Universidad de Oriente, Núcleo de Sucre, Cumaná, Venezuela Universidad de Oriente Posgrado en Biología Aplicada Universidad de Oriente Núcleo de Sucre Cumaná Venezuela; 2 Instituto de Investigaciones en Biomedicina y Ciencias Aplicadas, Universidad de Oriente Cumaná, Venezuela Universidad de Oriente Instituto de Investigaciones en Biomedicina y Ciencias Aplicadas Universidad de Oriente Cumaná Venezuela; 3 Centro de Investigaciones en Ciencias de la Salud, Universidad de Oriente, Núcleo de Anzoátegui, Barcelona, Venezuela Universidad de Oriente Centro de Investigaciones en Ciencias de la Salud Universidad de Oriente Núcleo de Anzoátegui Barcelona Venezuela

**Keywords:** Trypanosoma cruzi, enfermedad de Chagas/diagnóstico, recién nacido, ensayo de inmunoadsorción enzimática, Venezuela, Trypanosoma cruzi, Chagas diseases/diagnosis, newborn, enzyme-linked immunosorbent assay, Venezuela

## Abstract

**Introducción.:**

*Trypanosoma cruzi* se transmite principalmente por vía vectorial, sin embargo, las rutas oral y congénita han tomado relevancia.

**Objetivo.:**

Evaluar la infección por *T. cruzi* en mujeres puérperas y sus neonatos en el Hospital Universitario Dr. Luis Razetti de Barcelona, estado Anzoátegui, Venezuela.

**Materiales y métodos.:**

Se hizo un estudio prospectivo de corte transversal, de mayo de 2015 a agosto de 2016, en el que se evaluaron 1.200 mujeres para determinar la infección mediante las pruebas ELISA, MABA e IFI. Los neonatos de las madres seropositivas se evaluaron con la prueba de PCR y por serología a los nueve meses de edad. Se estimó la prevalencia de la infección por *T*. *cruzi* en mujeres puérperas y sus neonatos. Para establecer los factores de riesgo asociados a la infección, se usó la prueba de ji al cuadrado (c2) y la razón de probabilidad (OR).

**Resultados.:**

En total, 78 (6,50 %) mujeres resultaron positivas (IC95% 5,10-7,89 %). En seis (9,09 %) recién nacidos de madres seropositivas, se detectó ADN parasitario. Tras nueve meses de nacidos, once lactantes evaluados resultaron serológicamente negativos. La infección estuvo asociada con la duración del embarazo (OR=0,36; IC95% 0,15-0,84), origen del domicilio actual (OR=0,34; IC95% 0,24-0,62) o previo (OR=2,50; IC95% 1,38-4,52) y el tener familiares con la enfermedad de Chagas (OR=1,75; IC95% 1,02-3,01).

**Conclusiones.:**

La seroprevalencia para la infección por *T*. *cruzi* en mujeres puérperas del medio rural, fue elevada. La detección de ADN parasitario al momento del nacimiento no es indicativa de enfermedad de Chagas congénita.

## Abstract (en)

**Introduction.:**

*Trypanosoma cruzi* is mainly transmitted by vectors. Other pathways such as oral and congenital transmission have become increasingly relevant.

**Objective.:**

To evaluate *T*. *cruzi* infections in post-partum women and their newborns who attended the *Hospital Universitario Dr. Luis Razetti* (Barcelona, Anzoátegui state, Venezuela).

**Materials and methods.:**

A prospective cross-sectional study was undertaken from May, 2015, to August, 2016. ELISA, MABA and IFI assays were used to determine the infection in 1,200 post-partum women. The newborns of seropositive women were then examined for *T*. *cruzi* by PCR amplification and serological tests at nine months old. The prevalence of the parasitic infection in post-partum women and their newborns was then estimated. To establish the relationship between risk factors and infection, the chi-square test (c2) and the probability ratio (OR) was applied.

**Results.:**

A total of 78 women were identified as seropositive (6.50 %) (CI 95%: 5.10-7.89%), and parasitic DNA was detected in six of their newborns (9.09%). Nine months after birth eleven infants were examined, and all were found to be serologically negative. Risk factors detected were pregnancy duration (OR: 0,36; CI95%: 0,15-0,84), where the patients lived at present (OR: 0,34; CI95%: 0,24-0,62) or previously (OR: 2,50; CI95%: 1,38-4,52) and having relatives with Chagas disease (OR: 1,75; CI95%: 1,02-3,01).

**Conclusions.:**

Seroprevalence for *T*. *cruzi* infection in young post-partum women in rural areas was high. The detection of parasite DNA at birth was not indicative of congenital Chagas disease.

La enfermedad de Chagas es causada por el parásito protozoario *Trypanosoma cruzi*, el cual infecta entre seis y siete millones de personas en el mundo, de las cuales, cinco millones se encuentran en Latinoamérica. Actualmente, se estima que existen cerca de 70 millones de personas que están en riesgo de infectarse con el parásito ^1^.

La principal forma de transmisión de la infección por *T*. *cruzi* es la vectorial; sin embargo, existen otras como las transfusiones sanguíneas, el consumo de alimentos contaminados con orina y heces de triatominos infectados por el parásito, los trasplantes de órganos, los accidentes de laboratorio y la transmisión congénita. Esta última ha tomado gran importancia, tanto en los países latinoamericanos endémicos donde la parasitosis persiste, como en los países no endémicos en los que la migración de mujeres infectadas en edad fértil ha aumentado las cifras de infección congénita, colocándola por encima de las transfusiones de sangre o los trasplantes de órganos ^2^.

La transmisión materno-fetal de *T. cruzi* ocurre en una media del 5 %, sin embargo, existen diferencias entre las prevalencias reportadas en las áreas endémicas ^3^. En este sentido, según los últimos datos aportados por la Organización Mundial de la Salud (OMS), México presenta el mayor número de casos por año (1.788) de transmisión congénita del parásito, seguido por Argentina (1.457) y Colombia (1.046). Otros países endémicos, como Bolivia y Brasil, presentan 616 y 571 casos, respectivamente. En Venezuela, se reporta más de un millón de personas en riesgo de infectarse por *T*. *cruzi*; además, existen entre 100.000 y 200.000 personas ya infectadas, de las cuales 40.223 son mujeres en edad fértil (15 a 44 años). Venezuela se encuentra ubicada en el cuarto lugar en Latinoamérica con respecto al número de casos (665) con transmisión congénita por *T. cruzi*^1^.

Para hacer el diagnóstico de transmisión vertical de *T. cruzi*, primero debe confirmarse la infección de la madre mediante la detección de anticuerpos específicos de *T. cruzi* con dos pruebas serológicas con distinto principio, como el análisis inmunoenzimático de adsorción (ELISA), la *inmunofluorescencia* indirecta (IFI) y la hemaglutinación indirecta (HAI). Después de la confirmación de la infección de la madre, el parásito debe buscarse en el recién nacido mediante pruebas parasitológicas (microhematocrito, hemocultivo) y métodos moleculares, como la prueba de reacción en cadena de la polimerasa (PCR). Después de los nueve meses de edad, cuando los anticuerpos IgG transferidos por la madre han desaparecido, se realizan las pruebas serológicas. Por lo tanto, la detección del parásito mediante pruebas parasitológicas o la identificación de anticuerpos de tipo IgG anti-*T*. *cruzi* a los nueve meses de vida, es indicativa de transmisión congénita y el lactante debe ser tratado ^3^.

En Venezuela, son escasos los trabajos sobre la infección por *T. cruzi* en mujeres embarazadas, o puérperas, y la transmisión congénita. Por lo tanto, el objetivo del presente estudio fue obtener datos epidemiológicos relevantes sobre la situación actual de la infección por *T. cruzi* en mujeres puérperas y sus neonatos, atendidos en el Hospital Universitario Dr. Luis Razetti de Barcelona, estado Anzoátegui, así como también, conocer los factores de riesgo epidemiológicos asociados a la infección por *T. cruzi* en ese grupo poblacional de la región nororiental de Venezuela.

## Materiales y métodos

### Área del estudio

**Figura 1.**
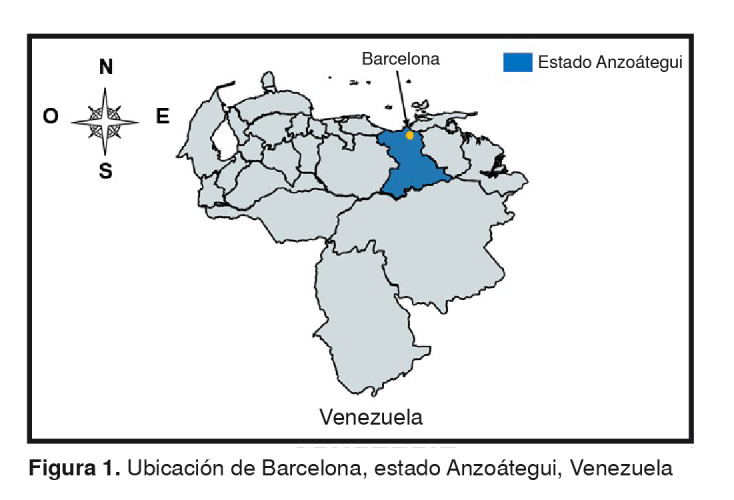
Ubicación de Barcelona, estado Anzoátegui, Venezuela

Este estudio se llevó a cabo entre mayo de 2015 y agosto de 2016 en el Hospital Universitario Dr. Luis Razetti de Barcelona, capital del estado Anzoátegui. Barcelona está ubicada a 10°08'00" de latitud norte y 64°41'00" de longitud oeste (figura 1). El Hospital está clasificado como de tipo IV. Es el principal centro hospitalario de la región y atiende las necesidades de los habitantes de la zona metropolitana, del resto del estado Anzoátegui y de los estados circunvecinos.

### Diseño del estudio y selección de la muestra

Se hizo un estudio prospectivo de corte transversal. El tamaño de la muestra se calculó de acuerdo con un estimado de 8.400 mujeres embarazadas por año que acudieron al centro hospitalario; se estableció un margen de error de 3 %, una prevalencia de 3 % y un intervalo de confianza de 95 % ^4^. En total, se evaluaron 1.200 mujeres puérperas y sus neonatos.

Se seleccionaron de manera aleatoria a las mujeres en puerperio fisiológico, es decir, en el periodo de 6 a 8 semanas que sigue al parto, en el cual las alteraciones fisiológicas y anatómicas producidas en el organismo materno durante el embarazo han vuelto al estado no gestante ^5^, que cumplían los siguientes criterios de inclusión:

- aceptación de la mujer puérpera para participar en la fase de diagnóstico de la infección por *T*. *cruzi*,
- aceptación de la participación de su neonato,
- aceptación de la participación en un estudio de seguimiento del neonato, en caso que la madre resultara seropositiva, y
- firma del consentimiento informado.

**Figura 2.**
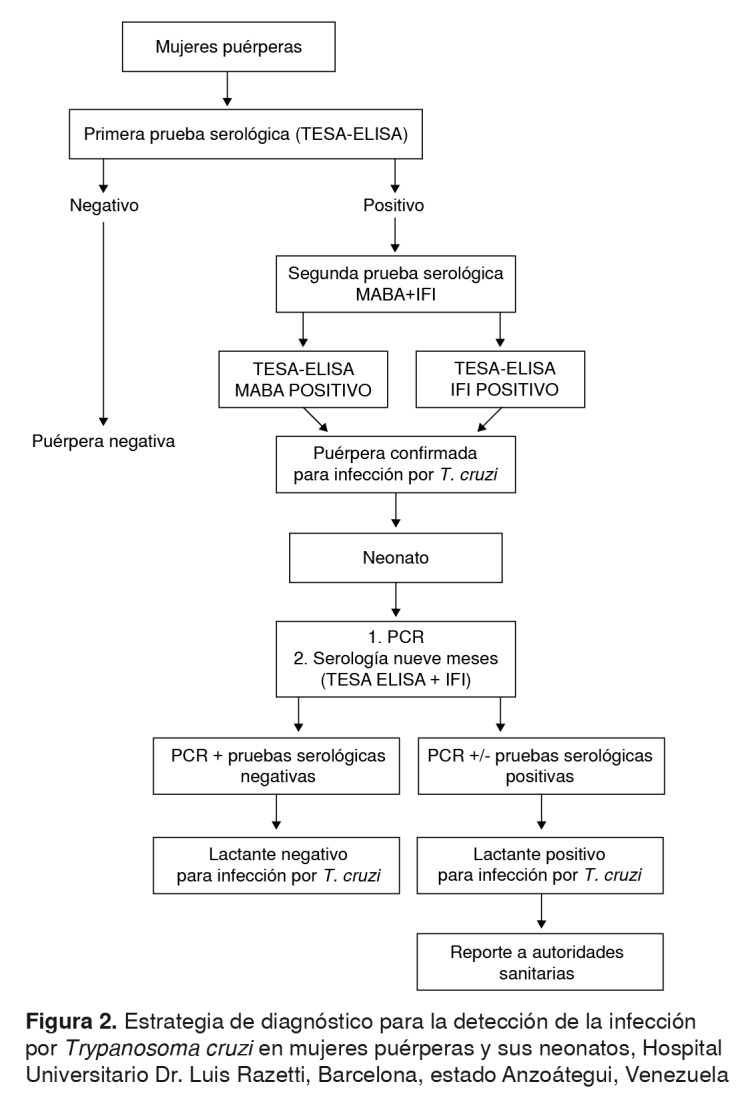
Estrategia de diagnóstico para la detección de la infección por *Trypanosoma cruzi* en mujeres puérperas y sus neonatos, Hospital Universitario Dr. Luis Razetti, Barcelona, estado Anzoátegui, Venezuela

Para la detección de los anticuerpos anti-*T. cruzi* en las muestras de los sueros de madres puérperas, se empleó la prueba ELISA, utilizando antígenos excretados o secretados de tripomastigotes de *T*. *cruzi* (TESA, *Trypomastigote Excreted-Secreted Antigens*). Todas las muestras positivas por TESA-ELISA se procesaron con la prueba de unión de múltiples antígenos (*MABA, Multiple Antigen Binding Assay)*, empleando TESA de *T*. *cruzi* (MABA-TESA) y con *inmunofluorescencia* indirecta, utilizando epimastigotes fijados de *T. cruzi* (aislado MHOM/VE/08/AU) ^6^. Los sueros de puérperas se consideraron positivos, cuando eran reactivos, por lo menos, con dos de las tres técnicas utilizadas (figura 2).

Los neonatos de las madres seropositivas se evaluaron mediante la prueba de PCR para la detección de ADN satélite de *T. cruzi* (PCRsat) ^7^. Transcurridos nueve meses después de su nacimiento, se reevaluaron mediante la prueba TESA-ELISA y por IFI para detectar anticuerpos anti-*T*. *cruzi* de tipo IgG (figura 2).

### Toma de muestra de sangre de las madres puérperas y sus lactantes

A cada participante se le extrajo por venopunción una muestra de sangre de 5 ml, aproximadamente. Para la separación del suero se vertió la muestra en un tubo sin anticoagulante y al cabo de media hora se procedió a la centrifugación. Todas las muestras de suero debidamente identificadas, se conservaron a una temperatura de -20 °C, hasta el momento de la realización de las pruebas serológicas. Cuando hubo necesidad de hacer la evaluación serológica de los lactantes, se procedió con la toma de muestra de manera similar a la descrita en el caso de las madres.

### Toma de muestra de sangre en los neonatos empleando papel de filtro

A los neonatos se les tomó una muestra capilar del talón, usando un dispositivo mecánico empleado habitualmente para la medición de la glucemia en los pacientes diabéticos. Después de un ligero masaje en el talón para estimular el flujo sanguíneo hacia la zona, se hizo la punción, previa asepsia del área con alcohol antiséptico. Se descartó la primera gota de sangre y, seguidamente, se recolectó la muestra sobre un círculo de papel de filtro Watmann N° 2. Las muestras se almacenaron individualmente en bolsas plásticas de cierre hermético identificadas con los datos del neonato y la fecha de la recolección, y se mantuvieron conservadas a -20 °C hasta el momento de practicar las pruebas moleculares.

### Prueba ELISA usando los antígenos de excreción o secreción de tripomastigotes de T. cruzi

Para el desarrollo de esta prueba, se utilizaron los antígenos de excreción o secreción de tripomastigotes de *T. cruzi* (cepa Tulahuen), siguiendo el procedimiento descrito por Berrizbeitia, *et al.*^8^*.* La dilución de trabajo para el anticuerpo primario y los controles, fue de 1:800 y, la del anticuerpo secundario, de 1:45.000 (Perkin-Elmer LifeScience™, Boston, MA).

Todos los sueros fueron evaluados por duplicado y en experimentos independientes. En cada placa, se incluyeron mezclas de sueros controles positivos y negativos, confirmados por tres pruebas serológicas diferentes en el Laboratorio de Inmunodiagnóstico de Chagas (Maracay, Venezuela).

Los resultados se aceptaron solo si el coeficiente de variación para cada placa era de 15 % o menos; de otro modo, las muestras eran analizadas nuevamente. El punto de corte para esta prueba se determinó utilizando la curva ROC (*Receiver-Operating Characteristic Curve*). Con esta curva se determinó el valor de 0,400 de densidad óptica como el valor óptimo, el cual permitió la discriminación entre los valores positivos y los negativos.

### Prueba de unión de múltiples antígenos

La transferencia de los antígenos a la membrana de nitrocelulosa (0,45 μm, Hybond, Amersham Biosciences, GE) se hizo incubando por una hora a temperatura ambiente 1 μg/ml de TESA de *T*. *cruzi* (cepa Tulahuen) en solución tampón de carbonato a pH 9,6 en dos líneas paralelas, usando un Miniblotter 28SL™ (Immunogenetics, Cambridge, MA, USA).

La membrana se lavó cinco veces con solución tampón de fosfato de sodio (pH 7,4) con 0,05 % de Tween 20 (PBS-Tween) y, finalmente, se bloqueó por incubación durante una hora a temperatura ambiente en una solución al 5 % de leche descremada y 0,1 % de Tween 20 (solución bloqueadora). Después, se cortó en tiras de 4 mm de ancho por 2 cm de largo. Estas se incubaron por dos horas a temperatura ambiente, con agitación suave en canales independientes (Mini Incubation Tray™, BioRad) con 800 μl de los sueros diluidos, 1:800 en solución bloqueadora.

Las tiras se lavaron ocho veces con PBS-Tween y se incubaron por dos horas a temperatura ambiente con agitación suave con 1 ml de anticuerpo secundario anti-IgG humana conjugado a peroxidasa (PerkinElmer LifeScience™, Boston, MA) diluido (1:32.000). Después de ocho lavados con PBS-Tween, se añadió a cada canal 1 ml de una solución de 3,3 diaminobencidina que contenía 0,1 % de peróxido de hidrógeno (Sigma Chemical Co.); las tiras se incubaron por 10 minutos a temperatura ambiente para permitir el desarrollo del color ^9^.

### Prueba de inmunofluorescencia indirecta

La prueba de IFI se practicó siguiendo el procedimiento descrito por Berrizbeitia, *et al*. ^6^. Brevemente, los epimastigotes fijados con formaldehído al 2 %, se colocaron de forma alterna en una lámina portaobjeto dividida previamente en ocho cuadrículas con marcador indeleble, y sobre cada una de estas se repartió el antígeno de *T*. *cruzi* (15 μl) diluido 1/16.

Las láminas se dejaron secar a temperatura ambiente por 12 horas, y se procedió a fijar la preparación con acetona fría por 10 minutos. Luego, en cada cuadrícula que contenía el antígeno, se colocaron 50 μl del anticuerpo primario diluido 1/32 hasta 1/1.024, sobre el área de la cuadrícula correspondiente en las láminas portaobjeto. Se colocaron las láminas en una cámara húmeda, y se llevaron a incubar a 37 °C durante una hora. Posteriormente, las láminas se lavaron dos veces consecutivas con solución PBS con pH 7,6, se secaron y se les agregó 50 μl de anti-IgG humana conjugada con fluoresceína (Sigma, USA), diluido 1/32 en PBS (pH 7,6), y se incubaron a 37 °C por una hora. Luego, se lavaron dos veces más.

Posteriormente, se agregaron 50 μl de la solución diluida de azul de Evans (1:1.000) en cada pocillo, se dejó durante cinco minutos a temperatura ambiente, y se enjuagaron las láminas con agua destilada. Se colocó una gota de glicerina tamponada y, sobre ella, una laminilla cubreobjetos. Las láminas se analizaron bajo el microscopio de fluorescencia (Zeiss) utilizando un filtro banda azul de 470-490 nm. La clasificación de la muestra como positiva o negativa obedeció a la visualización o no visualización de parásitos con emisión de fluorescencia (verde manzana). 

### Amplificación del ADN satélite de T. cruzi

La prueba se realizó según las condiciones descritas previamente ^7^. Brevemente, el ADN total fue extraído de las muestras de sangre recolectadas sobre papel de filtro, siguiendo las instrucciones del inserto de la casa comercial Qiagen. Para ello, se extrajo el ADN de las muestras sobre papel de filtro, para lo cual se recortaron tres círculos de 3 mm de diámetro del área manchada con sangre, cada uno equivalente a 0,5 μl aproximadamente.

Las muestras se colocaron en tubos Eppendorf de 1,5 ml de capacidad, con 180 μl de la solución tampón de desintegración, y se incubaron a 85 °C por 10 minutos. Pasado el tiempo, se centrifugaron los tubos a máxima velocidad y se agregó un volumen de 20 μl de proteinasa K, se agitó en un mezclador de vórtice (*vortex mixer*) y se volvieron a incubar los tubos a 56 °C por una hora. Posteriormente, se agregaron 200 μl de la solución tampón precipitante de proteína, se mezcló cada tubo con el mezclador de vórtice y se incubaron a 70 °C por 10 minutos.

Después de centrifugar a máxima velocidad, se retiraron los círculos de papel de filtro, se agregaron 200 μl de etanol a la fase fluida y la mezcla se aplicó a la columna del estuche Qiagen. Las reacciones de amplificación del ADN se hicieron empleando los oligonucleótidos TCZ3 (5’-TGCACTCGGCTGATCGTTT-3’) y TCZ4 (5’-TTCCTCC AAGCAGCGGATA-3’), que generan la amplificación de tres fragmentos de 168 pb, 360 pb y 550 pb del ADN satélite de *T*. *cruzi*.

La amplificación del ADN en la muestra se hizo en un volumen final de reacción de 50 μl. Las condiciones de reacción fueron: 2 U de *Taq* ADN polimerasa, 2 μM de MgCl2, 200 μM de desoxinucleótidos (dNTP), 1 μM de cada uno de los cebadores y 20 μl del ADN purificado. La amplificación se llevó a cabo en un termociclador (Gene Amp, PCR System 9700™) con una desnaturalización inicial a 94 °C por un minuto, seguido de 29 ciclos de cambio de temperatura entre 94 °C por 30 segundos (desnaturalización), 54 °C por 30 segundos, 54 °C por 90 segundos (amplificación), 72 °C por 30 segundos y 72 °C por 90 segundos (elongación), y por último, una extensión final a 72 °C por 5 minutos y estabilización a 37 °C por 10 minutos ^10^.

Los productos de amplificación fueron separados por electroforesis en geles de agarosa al 2 % y visualizados mediante la tinción con bromuro de etidio.

### Determinación de factores de riesgo e infección por Trypanosoma cruzi en mujeres puérperas

Para determinar la asociación entre las variables epidemiológicas y la infección por *T*. *cruzi*, el personal entrenado para ello entrevistó a cada una de las mujeres puérperas participantes en el estudio, una vez culminada la toma de muestras. En las encuestas se evaluaron los siguientes factores: aspectos sociodemográficos, edad, duración del embarazo, tipo de parto, antecedentes familiares de enfermedad de Chagas, lugar de residencia actual, haber vivido en el medio rural y haber habitado en viviendas con paredes de bahareque, reconocimiento de los triatominos y conocimiento de la enfermedad de Chagas.

### Análisis estadístico

Para establecer los posibles factores de riesgo mediante el análisis de las variables epidemiológicas y de los resultados de las pruebas serológicas, se usó la prueba de ji al cuadrado con la corrección de Yates. Igualmente, se determinó la razón de probabilidades (*odds ratio*, OR), para evaluar la probabilidad de la ocurrencia de un evento en presencia o ausencia de un factor de riesgo, con sus intervalos de confianza de 95 %.

Se utilizaron los programas de computación Excel 2010 (Microsoft Corp., USA), SPSS™, versión 18 (IBM Corp., Armonk, EE.UU.), para el procesamiento y el análisis estadístico de los datos.

### Aspectos éticos

Para la realización del estudio se contó con la aprobación de la Dirección del Hospital y con los permisos de los Servicios de Ginecología y Obstetricia, y de Pediatría, así como también del Comité de Bioética del Hospital Universitario Dr. Luis Razetti (Barcelona, Venezuela). Cada paciente firmó un consentimiento de participación.

Una vez que la mujer puérpera fue confirmada como positiva por dos pruebas serológicas, se le informó el resultado y la necesidad de hacer el seguimiento de su bebé después del nacimiento.

## Resultados

En el presente estudio, se analizaron 1.200 muestras de suero provenientes de madres puérperas y se tomaron 1.208 muestras de sangre en papel de filtro de sus neonatos (8 partos múltiples y 1.192 partos normales), para la detección de la infección por *T*. *cruzi*. La edad promedio de las puérperas evaluadas y la de las infectadas fue de 23±6,21 años (rango: 12 a 43 años) (cuadro 1) y 23±6,07 años (rango: 15 a 43 años), respectivamente. El promedio de horas para la toma de muestras sanguíneas una vez terminado el parto, fue de 22±17,39 horas (rango: 1 a 124 horas). El peso promedio de todos los neonatos evaluados fue 2.972±455,35 g (rango: 1.410 a 5.370 g). El promedio de peso de los neonatos de madres seropositivas fue de 3.005±452,04 g (rango: 1.960 a 4.200 g).

**Cuadro 1:** Seroprevalencia de la infección por *Trypanosoma cruzi*, según la edad en las mujeres puérperas, Hospital Universitario Dr. Luis Razetti, Barcelona, estado Anzoátegui, Venezuela

Rango de edad	Positivas	Negativas	Prevalencia %
(años)			(IC_95%_)
	
12-22	43	619	3,58 (2,53-4,64)
23-33	30	411	2,50 (1,62-3,38)
34-43	5	91	0,42 (0,05-0,78)
Total	78	1.121	6,50 (5,10-7,89)

χ2: 0,437; grados de libertad: 2; valor de p: 0,804; IC95%: intervalo de confianza

### Diagnóstico serológico en mujeres puérperas y lactantes

El 9,96 % (n=83) de los sueros evaluados, resultaron positivos en la prueba TESA-ELISA, y el 93,08 % (1.117 puérperas) fueron negativos. Todas las muestras positivas en la prueba TESA-ELISA se analizaron mediante MABA e IFI (figura 2). Con la primera, MABA, 74 muestras resultaron positivas y nueve negativas, mientras que, con la IFI, 43 fueron positivas y 39 negativas. Con la combinación de las pruebas ELISA y MABA, se detectaron 74 sueros positivos y 9 negativos; de estos últimos, cinco resultaron negativos y cuatro positivos en la IFI.

Para determinar la seroprevalencia de la infección por *T. cruzi* en las mujeres puérperas, se tuvieron en cuenta los 74 sueros positivos por ELISA y MABA, y cuatros sueros que resultaron positivos por ELISA e IFI. Por lo tanto, la seroprevalencia de la infección por *T. cruzi* en las mujeres evaluadas en el Hospital Universitario Dr. Luis Razetti, utilizando dos pruebas de diagnóstico serológico como lo recomienda la OMS, fue de 6,50 % (IC95% 5,10-7,89 %) (78/1.200 * 100) (cuadro 2).

**Cuadro 2:** Seroprevalencia de la infección por *Trypanosoma cruzi* en mujeres puérperas en el Hospital Universitario Dr. Luis Razetti, Barcelona, estado Anzoátegui, Venezuela, durante los meses de mayo de 2015 a agosto de 2016

Pruebas serológicas		
TESA-ELISAa	TESA-MABAb	IFIc	
Sueros positivos	83	74	43
Sueros negativos	1.117	9	39
Total	1.200		
Prevalencia (%)	6,50 (IC95% 5,10-7,89)		
			

a TESA-ELISA: antígenos de excreción o secreción de tripomastigotes de *T. cruzi* empleados en el análisis inmunoenzimático de adsorción

b TESA-MABA: prueba de unión de múltiples antígenos empleando TESA de *T. cruzi*

c IFI: prueba de inmunofluorescencia indirecta

Después de los nueve meses de nacidos, a los lactantes hijos de madres seropositivas se les practicaron las pruebas TESA-ELISA e IFI. De estos 78 lactantes, 11 (14,10 %) fueron evaluados para la determinación de anticuerpos de tipo IgG anti -*T*. *cruzi,* resultando todos negativos para la infección por el parásito mediante las pruebas TESA y ELISA (promedio de la densidad óptica: 0,18±0,17), y la IFI (figura 2).

### Detección de ADN satélite de Trypanosoma cruzi en recién nacidos

Se extrajo el ADN de 66 muestras de sangre de neonatos, recolectadas horas después del nacimiento en papel de filtro para la detección de la infección por *T*. *cruzi*, empleando la PCRsat, doce muestras no se procesaron por ser su volumen insuficiente para extraer el ADN. 

**Figura 3.**
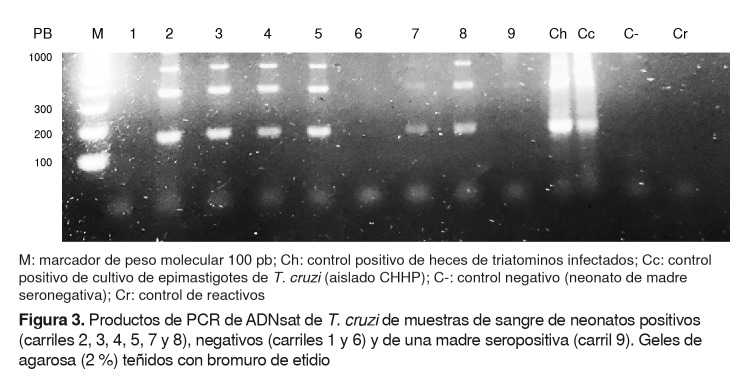
Productos de PCR de ADNsat de *T. cruzi* de muestras de sangre de neonatos positivos (carriles 2, 3, 4, 5, 7 y 8), negativos (carriles 1 y 6) y de una madre seropositiva (carril 9). Geles de agarosa (2 %) teñidos con bromuro de etidio

En la figura 3, se presenta un resumen de los resultados de la PCR de las muestras de sangre de neonatos eluídas de papel de filtro, que resultaron positivas en el procesamiento por PCRsat. Se muestra la amplificación de las bandas esperadas de 168 pb, 360 pb y 550 pb de ADN satélite de *T*. *cruzi* en seis neonatos (carriles 2, 3, 4, 5,7 y 8). Según estos datos, se detectó ADN de *T*. *cruzi* en 9,09 % ^6^ de los 66 neonatos evaluados por PCRsat. Cabe resaltar que tres de los seis neonatos positivos por PCRsat fueron negativos serológicamente después de los nueve meses de nacidos.

### Variables epidemiológicas

En este trabajo se evaluó la asociación de la infección por *T. cruzi* con diversas variables o factores de riesgo epidemiológico.

Se encontró una asociación estadística significativa entre la infección por *T*. *cruzi* y haber vivido en zonas rurales, la zona de residencia actual, tener familiares con la enfermedad de Chagas y la duración del embarazo. En total, 789 puérperas dijeron haber vivido previamente en una zona rural, de las cuales, 64 (8,10%) resultaron positivas para la infección por el parásito; mientras que, de las 411 pacientes que manifestaron haber vivido siempre en centros urbanos, 14 (3,40 %) fueron positivas para *T*. *cruzi*. Esta variable aumenta 2,5 veces el riesgo de infectarse con *T. cruzi*, por lo cual representa un importante factor de riesgo (OR=2,56). Esta asociación se mantuvo al evaluar las mujeres que aún residian en el área rural, encontrándose un mayor porcentaje de positivas (11,10 %) para la infección por *T. cruzi*, en comparación con las puérperas que habitaban en zonas urbanas (5,70 %). Asimismo, vivir en zonas urbanas resulta ser un factor protector contra lainfección por *T*. *cruzi* (OR=0,34; IC95% 0,24-0,62) (cuadro 3).

**Cuadro 3:** Factores de riesgo asociados a la infección por *T. cruzi* en mujeres puérperas, Servicio de Ginecología y Obstetricia, Hospital Universitario Dr. Luis Razetti, Barcelona, Anzoátegui

		Positivas para el					
Factor de riesgo	n	factor de riesgo		OR	^IC^ **95%**	p		
		% (n)					
							
Duración del embarazo							
A término	983	7,30	(72)	0,36	0,15-0,84	0,01*	
Prematuro	217	2,80	(6)				
Familiar con enfermedad de Chagas							
No	1.007	5,90	(59)	1,75	1,02-3,01	0,04*	
Sí	193	9,80	(19)				
Zona de residencia actual							
Rural	324	11,10	(36)	0,34	0,24-0,62	0,00*	
Urbana	873	5,70	(41)				
Ha vivido en zonas rurales							
No	411	3,40	(14)	2,50	1,38-4,52	0,002	
Sí	789	8,10	(64)			*	
Tipo de parto							
Natural	831	6,30	(52)	1,13	0,69-1,84	0,61	
Cesárea	369	7,00	(26)				
Transfusiones							
No	1126	6,40	(72)	1,31	0,55-3,12	0,54	
Sí	73	8,20	(6)				
Reconocimiento del vector							
No	655	6,10	(40)	1,15	0,72- 1,82	0,55	
Sí	545	7,00	(38)				
Conocimiento de la enfermedad de Chagas							
No	732	6,00(44)		1,22	0,77-1,94	0,39	
Sí	468	7,30	(34)				
Familiar fallecido por enfermedad de Chagas							
No	1109	6,50	(72)	1,01	0,42-2,40	0,97	
Sí	91	6,60	(6)				
Haber vivido en casa de bahareque							
No	743	5,70	(42)	1,42	0,90-2,26	0,12	
Sí	457	7,90	(36)				

OR: razón de proporciones (*odds ratio*); IC: intervalo de confianza; valor de probabilidad de la prueba de Fisher, p: probabilidad

* significativo p<0,05

Otro factor epidemiológico que se asoció con la infección por *T. cruzi*y aumenta el riesgo de adquirir la infección casi al doble (OR=1,75), fue tener familiares con la enfermedad de Chagas; así, de las 193 puérperas que manifestaron tener algún pariente con esta enfermedad, 19 (9,80 %) resultaron positivas para la infección por *T*. *cruzi*. Por otra parte, la mayoría de las 72 puérperas seropositivas presentó un embarazo a término (p<0,05), lo cual demostró en la presente investigación que estar infectado por *T*. *cruzi* no constituye un factor de riesgo para un parto prematuro (OR=0,36; IC95% 0,15-0,84) (cuadro 3).

Además, no se encontró una asociación estadística significativa entre haber tenido un parto natural o una cesárea, el conocimiento sobre la enfermedad de Chagas, el reconocimiento del vector, tener familiares fallecidos a causa de la enfermedad de Chagas, haber recibido transfusión sanguínea o haber vivido en casa de bahareque, con la infección por el parásito. Igualmente, no se encontró asociación entre la infección y la edad de las mujeres evaluadas**.**

## Discusión

La prevalencia de la infección por *T*. *cruzi* en las mujeres evaluadas fue de 6,50 %, la cual es significativamente mayor a la encontrada en una zona endémica de Perú (Arequipa), donde se reportó 0,73 % de seroprevalencia de esta parasitosis en 3.000 puérperas ^11^.

En cuanto a la infección por *T*. *cruzi* en mujeres embarazadas de otros países endémicos de Latinoamérica, su prevalencia es variable. Tal es el caso de un estudio de tres regiones de México (Ooxaca, Jalisco y Ciudad de México), en donde se obtuvo una prevalencia de 7,32 % para *T*. *cruzi* en mujeres embarazadas ^12^. Por el contrario, en los países de la subregión andina, como Colombia, y la región del Amazonas en el Ecuador, se reportaron prevalencias para esta parasitosis de 3,2 y 3,8%, respectivamente ^13^^,^^14^. En los países ubicados en Suramérica, la seroprevalencia de la infección por *T*. *cruzi* en mujeres embarazadas es mucho mayor, como la reportada en Argentina (Las Lomitas, Formosa: 29,1 %) y en Bolivia, que varía de 26,9 % (departamento de Santa Cruz) hasta 40,9 % (Yacuiba, región del Chaco) ^15^^-^^17^.

En relación con Venezuela, existen pocos estudios sobre la infección por *T*. *cruzi* en mujeres gestantes y la enfermedad de Chagas congénita, aunque la OMS reportó recientemente que ese país se encuentra en el cuarto lugar de aquellos con la mayor cantidad de casos anuales de este tipo de trasmisión ^1^.

La seroprevalencia de la infección por *T*. *cruzi* reportada en este trabajo, es mayor que la encontrada por Mastrolonardo, *et al*. ^18^, en 2013, quienes determinaron una prevalencia de 0,72 % (6 positivas) para la infección por *T. cruzi* en 828 embarazadas provenientes tanto del medio rural (Biscucuy, Portuguesa) como del urbano (Caracas). De los trabajos recientes en Venezuela en los cuales se menciona la transmisión congénita por *T*. *cruzi*, es importante destacar la demostración anatomopatológica del parásito por Alarcón de Noya, *et al.*^19^, en la autopsia de un feto de 24 semanas de gestación proveniente de una madre infectada por *T. cruzi*, posiblemente por vía oral.

Según las últimas investigaciones referentes a la enfermedad de Chagas congénita, la transmisión del parásito al feto depende de los siguientes factores: alta parasitemia en la madre, reacción inmunológica materna débil que conlleva aumento de la multiplicación del parásito; ruta de acceso del parásito al feto a través de áreas placentarias privadas de trofoblasto, y, por último, reacción inmunológica fetal insuficiente, tanto innata como específica, para controlar la multiplicación de los parásitos transmitidos ^3^.

Asimismo, aunque los marcadores moleculares actualmente utilizados para detectar las seis unidades discretas de tipificación (UDT) de *T. cruzi* (TcI a TcVI) no se centran en los genes responsables de la transmisión congénita o la capacidad patógena del parásito, todas las UDT han sido identificadas en casos humanos de infección congénita por *T. cruzi*, con excepción de TcIV. Por lo tanto, los diferentes genotipos de *T. cruzi* y sus características de población, como la capacidad patógena del parásito, la virulencia y el tropismo tisular, pueden jugar un papel importante en la infección congénita ^3^^,^^20^. Las características de las UDT de *T*. *cruzi* circulantes en la región nororiental de Venezuela ^21^, posiblemente determinan una baja transmisión del parásito durante el embarazo.

Para hacer el diagnóstico de infección por *T*. *cruzi* en madres puérperas y sus neonatos, en el presente estudio, se cumplió con los criterios señalados previamente ^22^. Al confirmarse la infección por *T. cruzi* en las madres seropositivas, se practicó una prueba molecular (PCRsat) en el recién nacido. Posteriormente, transcurridos nueve meses del nacimiento, se buscaron anticuerpos IgG específicos contra *T. cruzi* mediante pruebas serológicas. Por lo tanto, de acuerdo con los resultados, puede afirmarse que los 11 lactantes evaluados serológicamente después de nueve meses de nacidos fueron negativos para la infección por *T*. *cruzi.* De estos, en tres se había detectado ADN de *T. cruzi* en el momento del nacimiento mediante la prueba PCRsat. Por ello, debe considerarse la posibilidad de que el resultado positivo de la PCR en esos niños al momento del nacimiento, pueda obedecer a la transferencia de ADN no viable del parásito que cruza la placenta ^23^. Es por ello que se recomienda su repetición después de los nueve meses de vida de los infantes ^24^.

Los resultados obtenidos en la presente investigación corroboran que, aunque en diversas publicaciones la PCR ha demostrado sensibilidad y especificidad elevadas para el diagnóstico de la enfermedad de Chagas en la fase aguda, en el caso de la condición congénita, su uso aún resulta controversial. Campos, *et al*. ^25^ practicaron la PCR a 23 recién nacidos de madres infectadas, y obtuvieron amplificación de ADN de *T*. *cruzi* en nueve de ellos. Sin embargo, al transcurrir un año de su nacimiento, en seis niños no se encontraron anticuerpos anti-*T. cruzi* con una prueba comercial rápida (Chagas Stat-Pak™, Chembio Diagnostic Systems, Inc., IBM Company), ni con la prueba ELISA de tercera generación con antígeno recombinante (Accutrack, S.A. de C.V); estos resultados concuerdan con los obtenidos en la presente investigación.

Además, el éxito de la PCR depende de la cantidad de parásitos circulantes en el torrente sanguíneo de los pacientes ^26^. En la fase crónica, *T. cruzi* circula en cantidades muy pequeñas y las dinámicas sobre su circulación no son predecibles. Por lo tanto, una posible solución a esta limitación es la recolección de varias muestras de sangre, en serie y en diferentes momentos, o tomar un mayor volumen de sangre para cada prueba ^27^.

Entre las limitaciones del presente estudio, se puede mencionar que se evaluaron inicialmente las 1.200 mujeres puérperas para la detección de anticuerpos anti-*T*. *cruzi* únicamente mediante TESA-ELISA; en aquellas seropositivas, el diagnóstico se confirmó con otras pruebas serológicas. Sin embargo, la prueba TESA-ELISA ha demostrado gran sensibilidad (100 %) en el diagnóstico de la infección por *T*. *cruzi*^8^.

Otra limitación importante al efectuar este estudio, fue la dificultad para volver a contactar a las madres seropositivas, a fin de hacer el seguimiento de sus bebés y demostrar si hubo o no hubo transmisión congénita. Este mismo inconveniente ha sido reportado por otros investigadores, como Bern, *et al.*^28^, quienes llevaron a cabo un estudio para determinar la infección por *T. cruzi* en Santa Cruz (Bolivia), y solo pudieron hacer el seguimiento del 58 % de los bebes a los nueve meses de vida. En ese sentido, los autores plantean que esta situación conlleva la pérdida de la mitad de los casos estimados anualmente con infección congénita de *T. cruzi*.

Asimismo, De Rissio, *et al.*^29^, reconocen que, en el 55,8 % de los niños incluidos en su estudio para determinar la infección congénita del parásito en Buenos Aires (Argentina), no se logró completar el seguimiento durante los primeros doce meses de vida. Además, indican que este tipo de inconvenientes en el seguimiento de los posibles casos de transmisión vertical de *T. cruzi*, representa una vulnerabilidad que conlleva distorsión de la verdadera tasa de transmisión congénita. En la presente investigación, solo fue posible el seguimiento del 14 % de los lactantes de madres seropositivas.

En cuanto a las variables epidemiológicas evaluadas, se encontró asociación estadística significativa entre la infección por *T*. *cruzi*, y tener antecedentes de un familiar con la enfermedad de Chagas y vivir actualmente o haber vivido en una zona rural. Estos resultados son similares a los reportados por Orti, *et al.*^30^, quienes obtuvieron como factores estadísticamente significativos, la existencia de antecedentes familiares de tripanosomiasis y la procedencia rural, tras estudiar 383 mujeres gestantes, 37 de las cuales fueron positivas para la infección por *T. cruzi*. De ellas, 81,1 % habían vivido en zonas rurales (p=0,01) y 89,2 % tenían antecedentes familiares de la enfermedad de Chagas (p=0,01). Cucunubá, *et al.*^31^, también reportaron la asociación entre tener familiares con la enfermedad de Chagas y la infección por *T. cruzi*, en un estudio de 4.417 mujeres embarazadas, 119 de las cuales resultaron positivas mediante las técnicas ELISA e IFI. Igualmente, tras hacerles seguimiento a mujeres embarazadas en Casanare (Colombia), uno de los factores más relevantes relacionados con el riesgo de infección por *T. cruzi* fue la residencia en el medio rural (OR=2,2; IC95% 1,0-4,6) ^32^.

La infección por *T. cruzi* en mujeres embarazadas se asocia con frecuencia al aborto involuntario, el parto prematuro y el bajo peso al nacer ^33^. Sin embargo, en este estudio, el número de nacimientos prematuros fue menor que el de nacidos a término en mujeres seropositivas. Resultados similares obtuvieron Ávila, *et al.*^34^, tras realizar un cribado en 158 mujeres gestantes en Vizcaya (España); 19 mujeres gestantes presentaron infección por *T. cruzi*, de las cuales 15 tuvieron un parto a término sin trasmisión vertical del parásito.

Igualmente, Torrico, *et al.*^35^, compararon dos encuestas clínicoepidemiológicas sobre las consecuencias de la infección crónica de *T. cruzi* y la maternidad, en Cochabamba (Bolivia). Estos autores determinaron que, cuando no existe transmisión congénita del parásito y aunque la madre esté infectada, no se afectan la edad gestacional, el peso al nacer ni la salud general en los recién nacidos. Los resultados obtenidos en la presente investigación sustentan estas observaciones, ya que la mayoría de las mujeres puérperas seropositivas tuvieron un embarazo a término (OR=0,36; IC95% 0,15-0,84).

Aunque el haber vivido o continuar haciéndolo en condiciones precarias (casa de bahareque) y el tener contacto previo con el vector transmisor de *T*. *cruzi*, han demostrado ser factores de riesgo importantes en Argentina y Colombia ^13^^,^^30^^,^^36^, en esta investigación no se demostró asociación estadística significativa entre esas variables y la infección. Igualmente, no se encontró asociación entre la infección y la edad de las mujeres puérperas. El mayor número de puérperas seropositivas eran menores de 33 años. Estos resultados contrastan con los de un estudio en El Salvador; tras evaluar 1.506 mujeres embarazadas, en el que determinaron que aquellas con 35 o más años presentaban mayor riesgo de infección por el parásito (OR=3,54) ^37^.

Este es el primer estudio sobre la prevalencia de la infección por *T. cruzi* en mujeres puérperas y la posibilidad de transmisión al feto, llevado a cabo en el estado Anzoátegui, Venezuela. Se demostró una elevada prevalencia de esta parasitosis, en comparación con la encontrada en otros países de la subregión andina y en otros reportes de Venezuela. Aunque se detectó ADN del parásito en el momento del nacimiento en seis neonatos, solo tres de ellos fueron seguidos y resultaron negativos serológicamente después de los nueve meses de edad. Con los resultados obtenidos, no se pueden ofrecer cifras definitivas de transmisión congénita, ya que solo en tres casos fue posible descartar esta vía.

Los factores de riesgo identificados en este estudio (vivir o haber vivido en el medio rural, familiar con enfermedad de Chagas y duración del embarazo) y la elevada seroprevalencia, demuestran que deben implementarse medidas de control en las mujeres embarazadas, tales como incluir la detección de anticuerpos anti-*T. cruzi* entre las pruebas de rutina en ese grupo de pacientes y, así, reducir la probabilidad de transmisión de la enfermedad congénita.
